# Sorafenib as adjuvant therapy following radiofrequency ablation for recurrent hepatocellular carcinoma within Milan criteria: a multicenter analysis

**DOI:** 10.1007/s00535-022-01895-3

**Published:** 2022-07-11

**Authors:** Qunfang Zhou, Xiaohui Wang, Ruixia Li, Chenmeng Wang, Juncheng Wang, Xiaoyan Xie, Yali Li, Shaoqiang Li, Xianhai Mao, Ping Liang

**Affiliations:** 1 Department of Interventional Ultrasound, Chinese PLA General Hospital, 28 Fuxing Road, Beijing, 100853 China; 2 Department of Hepatobiliary Surgery, Hunan Provincial People’s Hospital, The First Affiliated Hospital of Hunan Normal University, Changsha, 410002 Hunan Province China; 3 Department of Liver Surgery, Sun Yat-Sen University Cancer Center, Guangzhou, 510060 Guangdong China; 4 Department of Medical Ultrasonics, Institute of Diagnostic and Interventional Ultrasound, The First Affiliated Hospital of Sun Yat-Sen University, Guangzhou, 510060 China; 5 Department of Liver Surgery, The First Affiliated Hospital of Sun Yat-Sen University, Guangzhou, 510060 Guangdong Province China

**Keywords:** Recurrent hepatocellular carcinoma, Sorafenib, Radiofrequency ablation, Milan criteria, Prognosis

## Abstract

**Background:**

Radiofrequency ablation (RFA) is considered as a convenient treatment with mild damage in treating recurrent hepatocellular carcinoma (RHCC). However, for patients with high risk of progression after RFA still needs new strategies to decrease the repeat recurrence.

**Methods:**

A total of 460 patients with RHCC within Milan criteria in four institutions were enrolled. 174 pairs were enrolled after propensity score matching (PSM). Overall survival (OS) and tumor-free survival (TFS) were compared between the two groups. A quantitative score system was established to screen out the beneficial population from RFA–sorafenib treatment.

**Results:**

The 1-, 3-, and 5-year OS rates were 97.7%, 83.7%, 54.7% for RFA–sorafenib group, and 93.1%, 61.3%, 30.9% for RFA group after PSM, respectively. Compared with the RFA group, the RFA–sorafenib group had significantly better OS (*P* < 0.001). The 1-, 3-, and 5-year TFS rates were 90.8%, 49.0%, 20.4% for RFA–sorafenib group, and 67.8%, 28.0%, 14.5% for RFA group after PSM. The difference was observed significantly between RFA–sorafenib group and RFA group (*P* < 0.001). A quantitative risk score system was established to precisely screen out the beneficial population from RFA–sorafenib treatment.

**Conclusions:**

Adjuvant sorafenib after RFA was superior to RFA alone in improving survival outcomes in patients with recurrent HCC within Milan criteria after initial hepatectomy. Subgroup analyses concluded that patients with high risk score had significantly longer survival from sorafenib administration.

**Supplementary Information:**

The online version contains supplementary material available at 10.1007/s00535-022-01895-3.

## Introduction

Hepatocellular carcinoma (HCC) is the fifth most common cancer and the third most common cause of cancer-related death worldwide [1]. Liver resection (LR) was recommended as first-line treatment for early staged HCC [2]. However, the long-term outcome of HCC after liver resection are not yet satisfactory, as the incidence of recurrence rate could be up to 60–80% within 5 years, and the reported 5-year survival rate of HCC ranges from 40 to 50% [2]. Approximately 30–50% of patients with recurrent HCC (RHCC) are diagnosed at an early stage [3]. Available treatment options for RHCC are almost the same as those for primary HCC. Currently, liver resection and ablation are available as the major curative treatments for early stage RHCC [2, 4–6]. RFA has the advantages of more repeat applications and fewer complications. Thus, RFA is considered safer with less damage in treating RHCC following primary resection [7]. A recent study reported that the 5-year overall survival rate of patients with RHCC within Milan criteria after receiving RFA treatment can be close to 40% [8]. Although the therapeutic effect of RFA has been confirmed, the prognosis of patients with RHCC still needs to be further improved. Thus, new strategies are required to decrease the repeat recurrence after RFA of RHCC.

Sorafenib is an oral multi-kinase inhibitor that suppresses tumor angiogenesis and proliferation by targeting serine/threonine kinases and receptor tyrosine kinases [9, 10]. Due to the high biological heterogeneity across HCC, Cheng et al. demonstrated that HCC patients with microvascular invasion (MVI) could benefit from adjuvant sorafenib after radical surgery [11]. Peng et al. proved that transarterial chemoembolization (TACE) combined with sorafenib was superior to TACE alone in improving survival outcomes of RHCC with MVI positive [12]. The main reasons for the satisfactory efficacy of sorafenib on HCC with MVI positive were that the presence of MVI indicated more aggressive behavior of the RHCC, and angiogenesis in RHCC with MVI positive was more abundant than the MVI negative [13].

On the contrary, the STORM trial indicated that sorafenib was not an effective intervention in the adjuvant setting for HCC following radical resection or ablation [14]. The underlying reasons may be explained by that most patients in the STORM trial (90%) had one lesion, less MVI positive and small tumor size, and these characteristics were signs of low risk of recurrence and low tumor aggressive behavior. On the other hand, Zhu et al. demonstrated that sorafenib combined with TACE and RFA in patients with medium or large (range, 3.1–7.0 cm in diameter) HCC were resulted in longer recurrence-free survival (RFS) and better overall survival (OS) than TACE combined RFA [15]. Feng et al. also proved that RFA combined sorafenib were associated with lower incidence of tumor recurrence rate and better survival than RFA alone in patients with primary HCC at early stage [16]. Sorafenib could inhibit tumor revascularization and blocking cell proliferation after ablation, resulting in longer RFS and OS of patients in the addition of sorafenib [17].

Although some studies have reported the efficacy of RFA combined with sorafenib on the treatment of primary HCC, its effect on RHCC has not been reported. It is well known that tumor recurrence is the most important factor affecting the long-term survival of patients, and timely, reasonable and appropriate treatment for patients with RHCC can further improve the long-term survival rate of patients with HCC [18]. Therefore, exploring new effective treatment methods, adopting reasonable treatment strategies, and effective treatment of RHCC are the key to improving the prognosis of patients. The aim of this study was to evaluate the efficacy of adjuvant sorafenib following RFA for the RHCC within Milan criteria and identified the relevant risk factors of survival and tumor progression.

## Patients and methods

### Patients

This multi-center study was conducted in patients with RHCC within Milan criteria from January 2009 to December 2015 at Chinese PLA General Hospital, Hunan Provincial People's Hospital, Sun Yat-sen University Cancer Center, The First Affiliated Hospital, Sun Yat-Sen University. The study was centrally approved by the ethics committee of these four centers and was conducted according to the guidelines of the Declaration of Helsinki [19]. Informed consent was waived, because this study was retrospective.

Eligibility criteria included clinical diagnosis of RHCC based on a history of partial hepatectomy for primary HCC. Patients who met the following criteria were enrolled: (1) 18–75 years; (2) patients who had recurrence for the first time after curative resection of primary HCC; (3) RHCC diagnosed by imaging studies (triphasic computed tomography and/or magnetic resonance imaging) showing both early enhancement and delayed decreased enhancement, in accordance with the American Association for the Study of Liver Diseases Practice Guideline for Management of HCC [20]; (4) RHCC met the Milan criteria, namely, single RHCC lesion less than 5 cm in diameter or no more than 3 tumors (each ≤ 3 cm in diameter) [21]; (5) patients with well-preserved liver function (Child–Pugh class A or B); (6) patients without any macroscopic invasion to the portal vein or metastasis to distant sites; and (7) RHCC without any history of local treatments, including radiofrequency ablation, interventional therapy et al. The excluding criteria were as follows: (1) under 18 years or over 75 years; (2) RHCC with tumor number > 3 or tumor diameter > 5 cm; (3) RHCC after radical thermal ablation; (4) RHCC with systemic therapy history (including molecular targeted therapy or immunotherapy); and (7) history of other malignancies; (8) incomplete clinical data.

### Outcomes and definitions

The primary endpoint for the study was overall survival (OS), and the secondary endpoint was tumor-free survival (TFS). OS was defined as the time from accepting RFA to death or last follow-up, and TFS was defined as the time from accepting the date of RFA to disease progression or last follow-up. The stage of RHCC recurrence was divided into early (≤ 2 years) and late recurrence (> 2 years) [22]. Hepatitis defined as a history of chronic hepatitis B virus (HBV) infection and/or positive hepatitis B virus RNA test. Cirrhosis was defined histologically by findings of initial resected liver specimens. Portal hypertension defined as esophageal varices and/or splenomegaly on imaging studies combined with a decreased platelet count [≤ 100 × 10^9^/L]). We used the albumin–bilirubin (ALBI) grade to evaluate liver function, because the ALBI grade is more accurate and objective than the conventional Child–Pugh score [23].

### Sorafenib administration

All patients in the Sorafenib–RFA group received RFA before accepting sorafenib. Sorafenib was recommended to patients met at least one of the following conditions: MVI positive, primary HCC > 5 cm, BCLC B stage of primary HCC state; early recurrent stage, multi-tumors of RHCC, RHCC larger than 3 cm. Sorafenib were given at an initial dosage of 400 mg twice daily without additional systemic therapies. Sorafenib was administered within 1 month after RFA, and patients received continual sorafenib. Drug-related complications were recorded. Sorafenib dose reduction was based on the presence of toxicity. If grade 3 or 4 hematologic toxicity, skin toxicity, gastrointestinal toxicity, hypertension, or hepatic dysfunction defined by National Cancer Institute Common Terminology Criteria for Adverse Events occurred [24], and a dose adjustment (400 mg once daily) was required until the adverse events were alleviated or eliminated. After dose adjustment, if grade 3 or 4 adverse events continued, sorafenib treatment was halted until the adverse effects were alleviated or until they disappeared.

### RFA procedure

RFA at each institution was performed by experienced physicians. Percutaneous RFA was performed using the cool-tip radiofrequency ablation system to achieve a single or multiple overlapping ablations with a goal to cover an area larger than the entire lesion plus an ablative margin of 0.5 cm or more. Ultrasound or contrast-enhanced ultrasound was used for tumor visualization. One or more single needles that can ablate 3.0–5.0 cm diameter volume at the highest energy setting. If imaging studies showed radiological features of residual tumor that suggested incomplete ablation in contrast enhanced ultrasound or CT, an additional session of percutaneous RFA with the intention of complete ablation was performed again. Complete ablation was defined as no area of enhancement was seen within or at the periphery of the ablated zone contrast enhanced CT or MRI.

### Follow-up

The follow-up period for this study was terminated on September 30, 2021. Patients were followed up once every 3 months for the 2 years after RFA and subsequently every 4–6 months. At each follow-up visit, alpha-fetoprotein (AFP) and liver function tests and abdominal ultrasonography or contrast enhanced CT scan or MRI was performed. Intrahepatic recurrence was defined as the appearance of one or more intrahepatic lesions with a longest diameter of at least 10 mm and a typical vascular pattern of HCC on dynamic imaging (enhancement in the arterial phase with washout in the portal venous or late venous phase). Lesions larger than 10 mm that did not show a typical vascular pattern could be diagnosed as HCC by evidence of a growth interval of at least 1 cm in subsequent follow-up. Extrahepatic recurrence was defined as new and growing lesions, especially multiple round nodules in the organs in imaging scans with elevation of serum AFP levels or not. Repeat RHCC was treated by further surgical resection, ablation, TACE, radiation therapy or systemic therapy according to the tumor recurrence status and the patient’s liver function.

### Propensity score matching (PSM) analysis

Propensity score-matching (PSM) analysis was used to reduce the effect of selection bias and potential confounding between the two groups. Propensity scores were estimated using a multivariate logistic regression model, by inserting the following variables: initial hepatectomy stage data (tumor diameter, tumor capsule, BCLC stage, MVI) and Recurrent stage data (sex, recurrent stage). Patients were matched 1:1 using the nearest neighbor method with a caliber of 0.10; and this matching process has been described in a previous study [25].

### Statistical analysis

To evaluate difference between the three groups, ANOVA was used to analyzed continuous variables, and the Pearson χ^2^ test and Fisher’s exact test were used to compare categorical variables. The survival curves OS and TFS were constructed according to the Kaplan–Meier method with the log-rank test, and the 1-, 2-, 3-, 4-, 5-year survival rates were determined using a life table using the *z* test. All statistical tests were 2 sides, and *P* < 0.05 was considered significant. The statistical analyses were performed using the Statistical Package for the Social Science (SPSS) software (version 22.0, SPSS Inc., Chicago, IL, USA) for Windows and R software for Windows (Version 3.6.4 http://www.r-project.org).

## Results

### Patient characteristics

A total of 2750 patients with HCC accepted curative resection from January 2009 to December 2015 at four centers. 1788 patients had recurrence and total of 460 patients were enrolled in this study. 185 patients received RFA combined sorafenib (RFA–sorafenib group) and 275 patients received RFA only (RFA group). Total of 254 tumors were ablated in the RFA–sorafenib groups and 371 tumors in RFA group. 7 tumors in RFA–sorafenib group were re-ablated and 12 tumors in RFA group. There were 174 pairs enrolled after PSM. The median follow-up was 62.0 months (range, 15–150 months) in the combination group and 60.9 months (range, 9–148 months) in the RFA group. Median duration of sorafenib was 14.8 months (range, 5.0–36.0 months). Compared to RFA group, patients in RFA–sorafenib group showed more patients with large (54.6% vs. 47.3%) and huge HCC proportion (23.2% vs. 17.8%), BCLC B stage (39.5% vs. 30.2%), MVI positive (46.5% vs. 33.8%), male proportion (78.4% vs. 66.9%), early recurrence proportion (69.2% vs. 56.4%). However, there were no significant differences between the 2 groups after PSM. The patient selection criterion was shown in Supplementary Fig. 1, and the demographic data, etiology of liver disease, and tumor characteristics of patients are summarized in Table 1.

**Table 1:** Baseline characteristics of patients who underwent radiofrequency ablation (RFA) or RFA–sorafenib for recurrent hepatocellular carcinoma (RHCC) within Milan criteria

Variable	Before PSM			After PSM		
	RFA–sorafenib 185 (*n*%)	RFA 275 (*n*%)	*P*	RFA–sorafenib 174 (*n*%)	RFA 174 (*n*%)	*P*
*Initial hepatectomy stage data*						
Surgical margin, cm						
≤ 1	140 (75.7)	217 (78.9)	0.415	134 (77.0)	137 (78.7)	0.698
> 1	45 (24.3)	58 (21.1)		40 (23.0)	37 (21.3)	
Tumor diameter, cm						
≤ 5	41 (22.2)	96 (34.9)	**0.012**	35 (20.1)	40 (23.0)	0.766
> 5, < 10	101 (54.6)	130 (47.3)		99 (56.9)	93 (53.4)	
≥ 10	43 (23.2)	49 (17.8)		40 (23.0)	41 (23.6)	
BCLC stage						
A	112 (60.5)	192 (69.8)	**0.039**	108 (62.1)	101 (58.0)	0.444
B	73 (39.5)	83 (30.2)		66 (37.9)	73 (42.0)	
Tumor capsule						
Incomplete	112 (60.5)	190 (69.1)	0.058	104 (59.8)	112 (64.4)	0.377
Complete	73 (39.5)	85 (30.9)		70 (40.2)	62 (35.6)	
MVI						
Negative	99 (53.5)	182 (66.2)	**0.006**	99 (56.9)	98 (56.3)	0.914
Positive	86 (46.5)	93 (33.8)		75 (43.1)	76 (43.7)	
Tumor differentiation						
I–II	112 (60.5)	176 (64)	0.452	107 (61.5)	110 (63.2)	0.740
III–IV	73 (39.5)	99 (36)		67 (38.5)	64 (36.8)	
Hepatitis						
Negative	57 (30.8)	88 (32)	0.788	52 (29.9)	55 (31.6)	0.727
Positive	128 (69.2)	187 (68)		122 (70.1)	119 (68.4)	
Cirrhosis						
Negative	88 (47.6)	125 (45.5)	0.656	82 (47.1)	81 (46.6)	0.914
Positive	97 (52.4)	150 (54.5)		92 (52.9)	93 (53.4)	
*Recurrent stage data*						
Age, years						
< 60	131 (70.8)	201 (73.1)	0.593	123	124 (71.3)	0.906
≥ 60	54 (29.2)	74 (26.9)		51	50 (28.7)	
Sex						
Male	145 (78.4)	184 (66.9)	**0.008**	135	129 (71.1)	0.452
Female	40 (21.6)	91 (33.1)		39	45 (25.9)	
HBV–DNA level, IU/mL						
< 1000	99 (53.5)	165 (60)	0.168	92	94 (54.0)	0.830
≥ 1000	86 (46.5)	110 (40)		82	80 (46.0)	
AFP, ng/mL						
< 200	96 (51.9)	164 (59.6)	0.100	88	101 (58.0)	0.162
≥ 200	89 (48.1)	111 (40.4)		86	73 (42.0)	
RHCC diameter, cm						
≤ 3	144 (76.2)	202 (73.5)	0.286	136	125 (71.8)	0.173
> 3	41 (23.8)	73 (26.5)		48	49 (28.2)	
RHCC number						
Single	129 (69.7)	192 (69.8)	0.984	123	120 (69.0)	0.726
Multiple	56 (30.3)	83 (30.2)		51	54 (31.0)	
ALBI grade						
I	136 (73.5)	187 (68)	0.205	127	120 (69.0)	0.408
II	49 (26.5)	88 (32)		47	54 (31.0)	
Recurrent stage						
Early	128 (69.2)	155 (56.4)	**0.006**	56	50 (28.7)	0.485
Late	57 (30.8	120 (43.6)		118	124 (71.3)	

The bold *P* values represent the significance between the two groups

BCLC, Barcelona Clinic Liver Cancer; RHCC, recurrent hepatocellular carcinoma; RFA, radiofrequency ablation; MVI, microvascular invasion; HBV, hepatitis B virus; AFP, alpha fetoprotein; ALBI, albumin–bilirubin

### Overall survival analysis

**Fig. 1:**
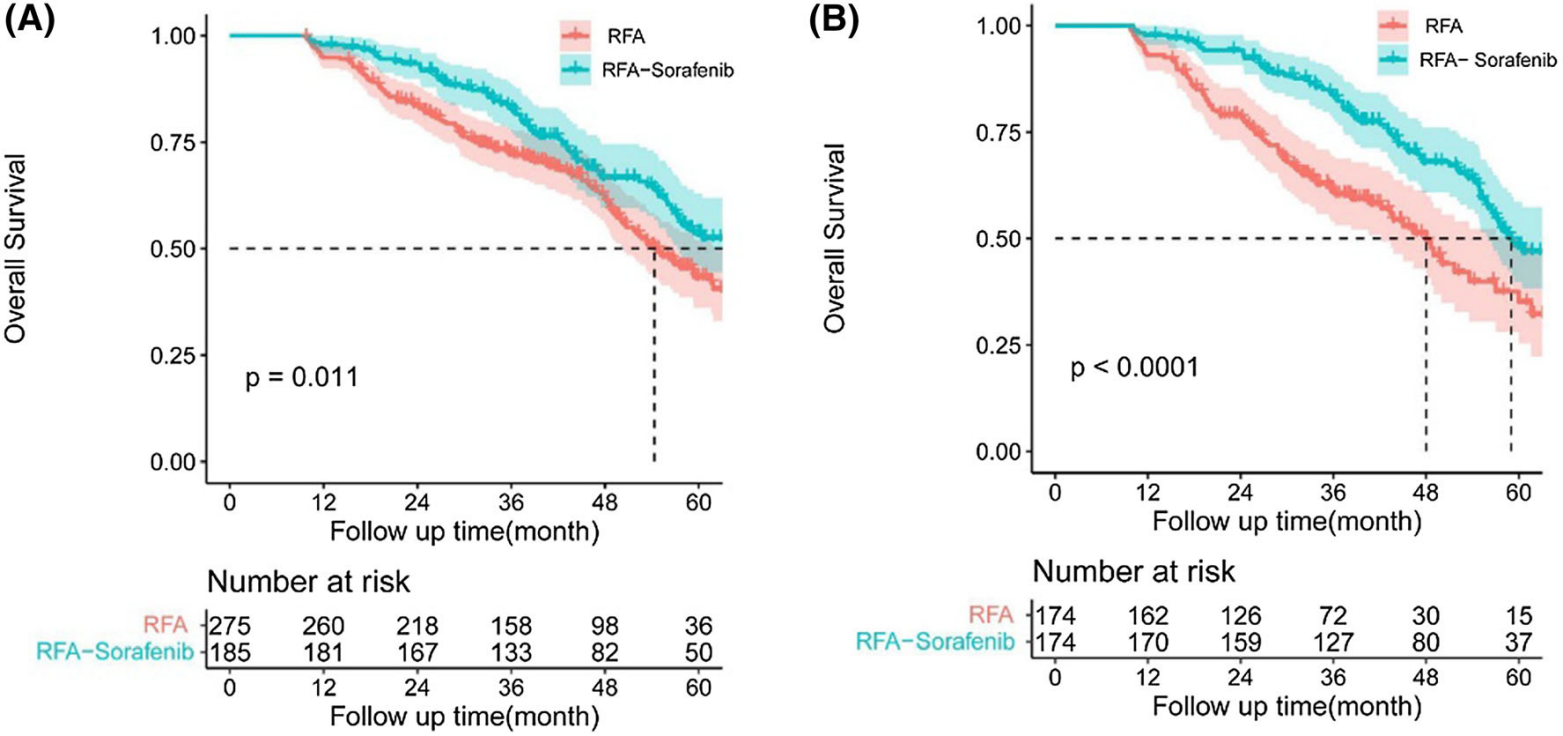
Kaplan–Meier curves for overall survival (OS) before (**A**) and **B** after propensity score matching (PSM) in patients with recurrent hepatocellular carcinoma (RHCC) within Milan criteria. The OS rates for patients who had RFA–sorafenib were significantly higher than those who had RFA (*P* = 0.011), and the OS rates after PSM was also significant after PSM (*P* < 0.001)

Before PSM, the median OS in RFA–sorafenib was 64.3 ± 4.0 months (95% confidence interval (CI) 56.4–72.2) vs. 56.3 ± 3.1 months (95% CI 50.3–62.4). The difference in OS was statistically significant between RFA–sorafenib group and RFA group (*P* = 0.011) (Fig. 1A). However, after PSM, the median OS was 65.2 ± 4.2 months (95% CI 57.1–73.4) in RFA–sorafenib group and 48.0 ± 2.7 months (95% CI 42.7–53.4) in RFA group. The difference in OS was statistically significant between RFA–sorafenib group and RFA group (*P* < 0.001) (Fig. 1B). The 1-, 3-, and 5-year OS rates were 97.7%, 83.7%, 54.7% for RFA–sorafenib group, and 93.1%, 61.3%, 30.9% for RFA group after PSM, respectively. (Table 2).

**Table 2:** Overall survival (OS) and tumor-free survival (TFS) rates of patients who underwent radiofrequency ablation (RFA) or RFA–sorafenib for recurrent hepatocellular carcinoma (RHCC) after propensity score matching (PSM)

	RFA–sorafenib (%)	RFA (%)	*P*
1-Year OS	97.7	93.1	0.018
2-Year OS	94.2	79.0	< 0.001
3-Year OS	83.7	61.3	< 0.001
4-Year OS	67.9	51.2	0.003
5-Year OS	54.7	30.9	0.001
1-Year TFS	90.8	67.8	< 0.001
2-Year TFS	70.1	44.3	< 0.001
3-Year TFS	49.9	28.0	< 0.001
4-Year TFS	24.7	15.1	0.033
5-Year TFS	20.4	14.5	0.136

Multivariate analysis after PSM revealed that RFA treatment (HR = 4.05; 95% CI 2.84–5.78; *P* < 0.001), AFP ≥ 200 ng/mL (HR = 1.12; 95% CI 1.06–1.80; *P* = 0.048), multiple tumors (HR = 1.94; 95% CI 1.42–2.69; *P* < 0.001), early recurrent stage (HR = 2.56; 95% CI 1.76–3.75; *P* < 0.001), primary HCC size ≥ 10 cm (HR = 1.571.5; 95% CI 1.17–2.52; *P* = 0.035), BCLC B stage (HR = 1.26; 95% CI 1.11–1.76; *P* = 0.042), MVI positive (HR = 1.43; 95% CI 1.04–1.96; *P* = 0.028) were associated with poorer OS (Table 3).

**Table 3:** Analysis of clinicopathological characteristics impacting overall survival (OS) in entire patients with hepatocellular carcinoma (RHCC)after propensity score matching (PSM)

	Comparison	Univariate analysis		Multivariate analysis	
		HR (95% CI)	*P*	HR (95% CI)	*P*
*Recurrent stage data*					
Age level, years	< 60 vs. ≥ 60	0.83 (0.59–1.17)	0.289		
ALBI grade	I vs. II	1.23 (0.93–1.79)	0.127		
HBV–DNA positive	No vs. yes	0.77 (0.57–1.05)	0.104		
Anti-virus	No vs. yes	0.83 (0.61–1.14)	0.249		
AFP level, ng/mL	< 200 vs. ≥ 200	**1.42 (1.04–1.94)**	**0.026**	**1.12 (1.06–1.80)**	**0.048**
Tumor size. cm	≤ 3 vs. > 3	1.11 (0.79–1.56)	0.548		
Tumor number	Single vs. multiple	**2.13 (1.54–2.95)**	** < 0.001**	**1.94 (1.42–2.69)**	** < 0.001**
Recurrent stage	Late vs. early	**2.72 (1.82–4.04)**	** < 0.001**	**2.56 (1.76–3.75)**	** < 0.001**
Types of treatment	RFA–Sorafenib vs. RFA	**2.18 (1.57–3.02)**	** < 0.001**	**4.05 (2.84–5.78)**	** < 0.001**
*Initial hepatectomy stage data*					
Tumor size, cm	≤ 5 > 5, < 10 ≥ 10	Reference 1.50 (0.97–2.32) **1.92 (1.18–3.12)**	0.057 **0.009**	**Reference** 1.29 (0.84–1.98) **1.57 (1.17–2.52)**	0.238 **0.035**
BCLC stage	A vs. B	**1.55 (1.12–2.15)**	**0.009**	**1.26 (1.11–1.76)**	**0.042**
MVI	Negative vs. positive	**1.58 (1.16–2.14)**	**0.004**	**1.43 (1.04–1.96)**	**0.028**
Resection margin, cm	> 1 vs. ≤ 1	0.72 (0.48–1.08)	0.111		
Tumor differentiation	I–II vs. III–IV	1.22 (0.89–1.67)	0.216		
Tumor capsule	Complete vs. incomplete	0.93 (0.79–1.09)	0.349		
Hepatitis	No vs. yes	0.77 (0.55–1.07)	0.115		
Cirrhosis	No vs. yes	0.97 (0.71–1.32)	0.822		

The bold *P* values represent the significance between the two groups

ALBI, albumin–bilirubin; AFP, alpha-fetoprotein; BCLC, Barcelona Clinic Liver Cancer; HR, hazard ratio; CI, confidence interval; MVI, microvascular invasion

### Tumor-free survival (TFS) analysis

**Fig. 2:**
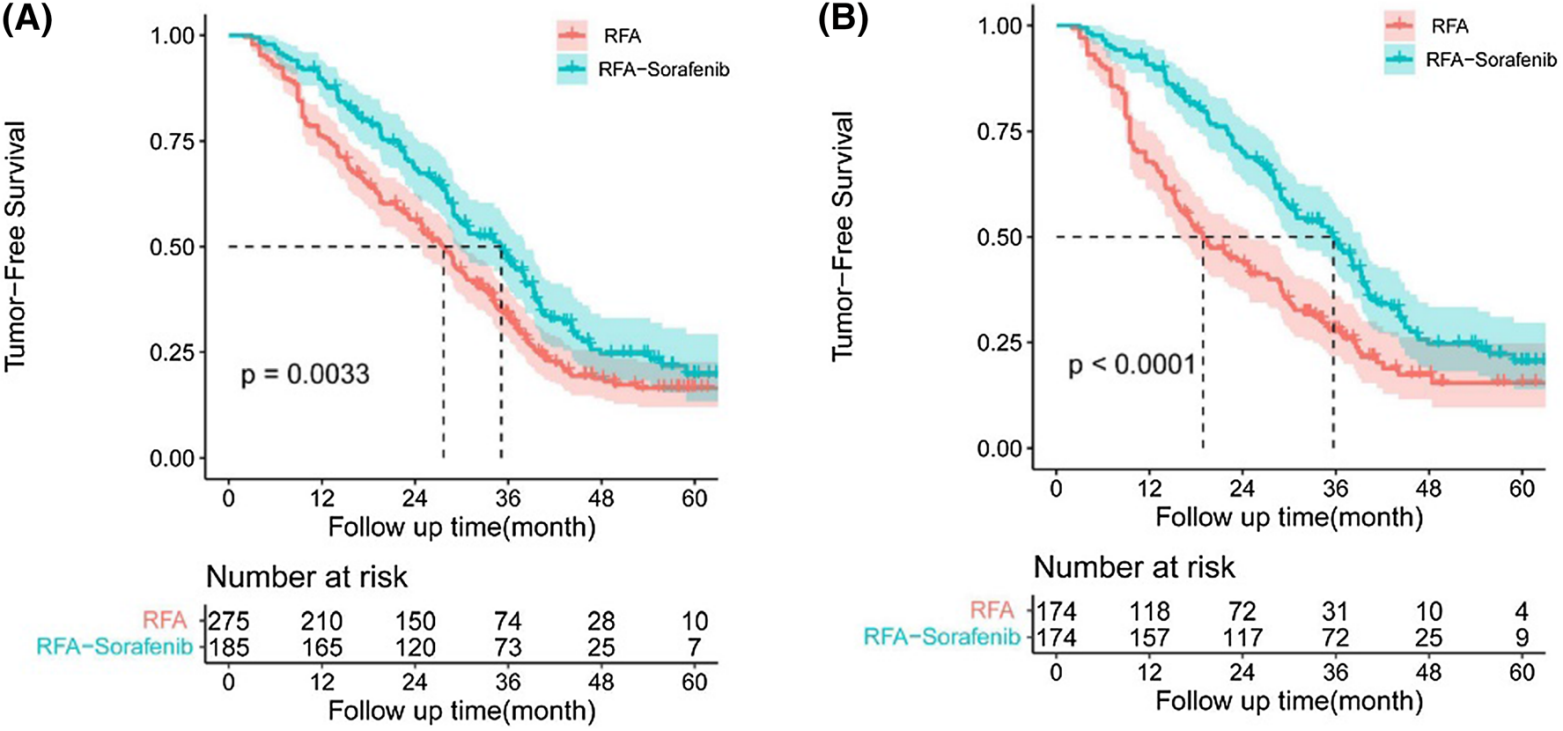
Kaplan–Meier curves for tumor-free survival (TFS) before (**A**) and **B** after propensity score matching (PSM) in patients with recurrent hepatocellular carcinoma (RHCC) within Milan criteria. The TFS rates for patients who had RFA–sorafenib were significantly higher than those who had RFA (*P* = 0.003), and the TFS rates after PSM was also significant (*P* < 0.001)

Before PSM, For and RFA groups, the median TFS was 35.1 ± 2.5 months (95% CI 30.3–39.9) in the RFA–sorafenib and 27.6 ± 1.3 months (95% CI 25.1–30.2) in RFA group. The difference was obvious between RFA–sorafenib group and RFA group (*P* = 0.003) (Fig. 2A). For the RFA–sorafenib and RFA groups after PSM, the median TFS was 35.7 ± 2.7 months (95% CI 30.4–41.0) vs. 18.9 ± 2.1 months (95% CI 14.8–23.0). The 1-, 3-, and 5-year TFS rates were 90.8%, 49.0%, 20.4% for RFA–sorafenib group, and 67.8%, 28.0%, 14.5% for RFA group after PSM, respectively. (Table 2). The difference was observed significantly between RFA–sorafenib group and RFA group (*P* < 0.001) (Fig. 2B). We further analyzed the repeat recurrence between the two groups. There was no difference of recurrence characteristics (Supplementary Table 1).

Multivariate analysis revealed that ALBI grade II (HR = 1.29; 95% CI 1.14–1.91; *P* = 0.007), multiple tumors (HR = 1.65; 95% CI 1.29–2.1; *P* < 0.001), early recurrent stage (HR = 2.22; 95% CI 1.75–2.85; *P* < 0.001), RFA treatment (HR = 1.52; 95% CI 1.22–1.89; *P* < 0.001), primary HCC size ≥ 10 cm (HR = 1.83; 95% CI 1.35–2.49; *P* < 0.001), BCLC B stage (HR = 1.38; 95% CI 1.09–1.73; *P* = 0.007), MVI positive (HR = 1.42; 95% CI 1.14–1.77; *P* = 0.002) were associated with poorer TFS (Supplementary Table 2).

### Subgroup analyses of OS

**Fig. 3:**
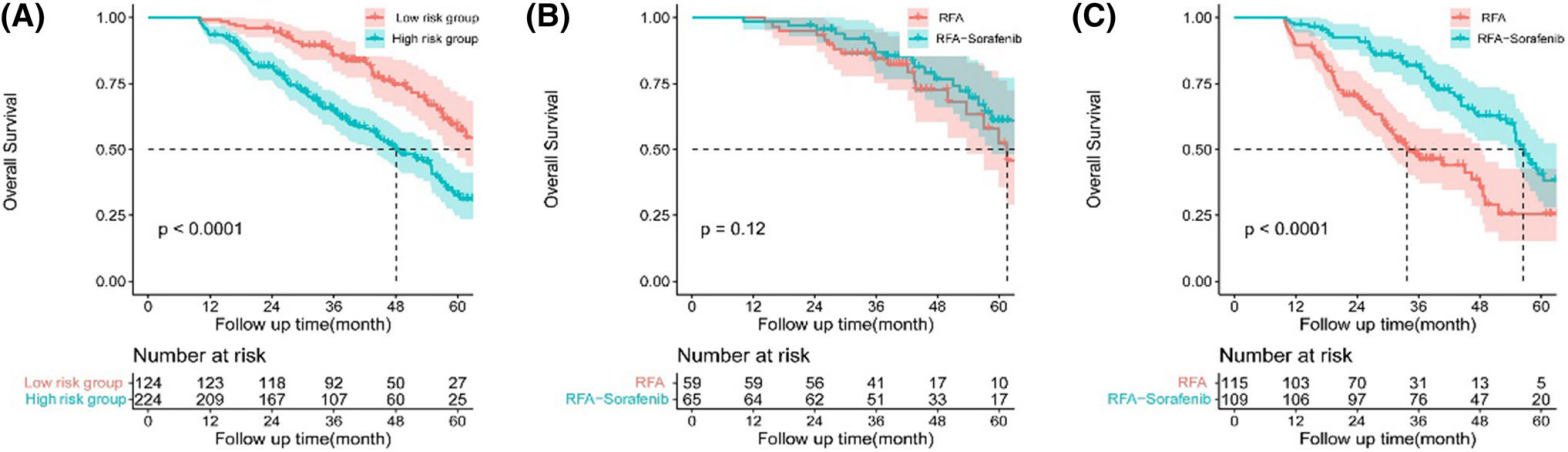
Kaplan–Meier survival curves for patients with different risk score. **A** OS curve of entire patients with low and high risk group. OS of Sorafenib–RFA and RFA in the low risk group (**B)** and in the high risk group (**C**). There was no difference between RFA or RFA–sorafenib treatment in the low risk group (*P* = 0.120), while there was significant discrepancy in the high risk group (*P* < 0.001)

To screen out which population of patients could benefit from the combination of RFA–sorafenib, we conducted subgroup analysis. We labeled a quantitative risk score for each factor which was significant in multivariate analysis of OS (Table 4). The risk score ranged from 6 to 13 points in the entire population, and patients were divided into two groups based on the risk score, namely, high risk (score ≥ 9) and low risk (score < 9). 224 (64.4%) patients were in high risk group and 124 (35.6%) patients were in low risk group. The OS was obviously different between the two groups (Fig. 3A, *P* < 0.001). 115 (66.1%) patients were with high risk score in RFA group, and 109 (62.6%) patients were with high risk score in RFA–sorafenib group. Multilevel of RFA and RFA–sorafenib comparison in the high risk and the low risk groups were analyzed, and the result revealed that no difference was observed between RFA or RFA–sorafenib treatment in the low risk group (Fig. 3B, *P* = 0.120), while there was significant discrepancy in the high risk group (Fig. 3C, *P* < 0.001). The TFS was obviously different between the low and high risk groups (Supplementary Fig. 2A). TFS were significant between RFA or RFA–sorafenib treatment in low risk group (Supplementary Fig. 2B) and high risk group (Supplementary Fig. 2C).

**Table 4:** Risk score weight of factors which were significant in multivariate analysis

Variables	Score		
	1	2	3
AFP level	< 200	≥ 200	
Tumor number	Single	Multiple	
Recurrent stage	Late	Early	
Primary tumor size, cm	≤ 5	> 5, < 10	≥ 10
BCLC stage	A	B	
MVI	Negative	Positive	

AFP, alpha-fetoprotein; BCLC, Barcelona Clinic Liver Cancer; MVI, microvascular invasion

### Sorafenib related adverse events

All of 185 patients in RFA–sorafenib received at least 3 months of sorafenib. The emergent adverse events with frequency higher than 5% were recorded (Supplementary Table 3). For the grades 1–2 of adverse events, patients alleviated after accepting symptomatic treatment or dose reduction. For the 3–4 grade level, patients were temporary stopped the sorafenib until the adverse effects were alleviated, and low dose of sorafenib were continued if possible after recovery.

## Discussion

The treatment of RHCC is an urgent and challenging clinical problem. At present, clinical consensus has been reached on the diagnosis and treatment of primary HCC and guidelines have been issued, but the choice of treatment for RHCC is still controversial [26]. Although the existing treatment methods including re-resection, ablation, interventional therapy, radiotherapy and chemotherapy, and targeted immunotherapy have achieved certain effects in RHCC, there is still no systematic treatment system for RHCC [18, 27]. Therefore, for RHCC with a higher risk of recurrence, it is of great significance to further explore new treatment models to reduce tumor recurrence and improve patient survival. In this multicenter study, we found that the combination of RFA–sorafenib provided more effective in improving overall survival and tumor-free survival than RFA only for RHCC within Milan criteria. To date, this is the first reporting efficacy of sorafenib on RHCC after radical RFA therapy.

It is well known that efforts to prevent tumor recurrence and provide appropriate management of RHCC are keys to improve the survival of patients. RFA with fewer complications compared with surgery has been commonly used to treat RHCC. However, there is no universally accepted form of adjuvant therapy for preventing recurrence after RFA. In addition, molecular targeted drug therapy for HCC has become a hotspot of clinical research, which can improve the anti-tumor ability of the body, effectively delay tumor progression time, reduce tumor recurrence and improve the long-time survival of patients [28]. Sorafenib as the first-line drug for the earliest targeted therapy, the safety and efficacy for patients with advanced HCC has been proved, and now it has been widely accepted for HCC therapy [29]. A series of studies have been reported that combination of RFA and sorafenib on primary HCC was safe and effective, controlling tumor progression and prolonging survival better than sorafenib or RFA alone [16, 30, 31]. However, there was no research in exploring combination of RFA and sorafenib on RHCC. Although the STORM study showed that sorafenib was not an effective intervention in the adjuvant setting for primary HCC after resection or ablation, RHCC differs from primary HCC including the genomic and epigenomic features [32, 33]. Therefore, exploring whether patients with recurrent HCC could benefit from sorafenib after radical RFA is essential to improve the prognosis of patients.

In our study, the results showed that the 1-, 3-, and 5-year OS rates were 97.7%, 83.7%, 54.7% for RFA–sorafenib group, and 93.1%, 61.3%, 30.9% for RFA group after PSM, respectively. Compared with the RFA group, the RFA–sorafenib group had significantly better OS (Fig. 1), which was consistent with those reported in previous studies of primary HCC (15, 16). The 1-, 3-, and 5-year TFS rates were 90.8%, 49.0%, 20.4% for RFA–sorafenib group, and 67.8%, 28.0%, 14.5% for RFA group after PSM, respectively. Similarly, patients in RFA–sorafenib group had obvious higher rates of TFS than RFA group (Fig. 2). 1-, 3-, and 5-year TFS rates in Feng et al. were 50.2%, 21.9%, 19.2% (4), and 85.0%, 52.4%, and 36.2% in the Xia et al. [8]. Although TFS of RFA in our study was a little different from the results in the Xia et al., it is acceptable due to the patients bias in different studies. More patients in our study were with higher proportion of large and huge HCC and more patients at BCLC B stage and higher MVI positive at initial hepatectomy baseline, and higher proportion of multi tumors at recurrent stage information than patients in Xia et al. it is well known that all the factors indicated poorer TFS and aggressive tumor behavior [34–36]. More importantly, our findings are the first to demonstrate that adjuvant administration of sorafenib after RFA significantly improves survival in patients with RHCC.

It is important to point out that majority of patients with multi-factors correlated with worse survival. Thus, a comprehensive risk score system to precisely pick out the patients with high risk of recurrence was essential. In our study, we labeled each factor which was relevant to OS a quantitative risk score. The risk score ranged from 6 to 13 points in the entire population, and the patients were divided into two groups based on the risk score. The higher was the risk score, the worse was the survival. Patients in the high risk group showed survival increase from the addition of sorafenib. There were 65 patients in the low risk group received adjuvant sorafenib after RFA treatment even though there was no significance in OS between the two groups. Sorafenib was recommend after RFA when patients met one of the risk factors, and these risk factors were all significant in OS between the two groups. While not all patients might benefit from the adjuvant sorafenib. Thus, it was vital to establish a risk score system to screen out the patients precisely and guide the drug administration. This risk score system could effectively guide the future treatment of RHCC within Milan criteria, and our study could be applied not for sorafenib but also other multi-kinase inhibitors, such as lenvatinib and donafenib, and other anti-angiogenesis drug-like bevacizumab. It is important to point out that the duration of patients received sorafenib varied. In this multicenter study, the doctors usually proposed patients to receive at least 1 year of sorafenib administration after RFA, and it would be stopped if the tumor progression occurred. For patients without progression after 1 year, sorafenib administration depended on the patients’ liver function, the doctor’s recommendation, patients’ choice and economic affordability. If severe adverse events occurred and continued, sorafenib treatment was also halted.

This study has some limitations. First, as a retrospective study, the selection bias existed in determining patients using sorafenib even though a PSM was used to balance the bias, because it was not only the choice of doctors but also the patient’s tolerance and affordability. Second, although we have carefully selected patients with several clinical characteristics, the influence of measured and unmeasured confounders on the outcome of patients is inevitable. For example, heterogeneous RFA modalities and doctor’s experience, and their combinations might make some sense to the outcome in some extent unknown. Third, future prospective study needed to verify this funding which could be as a guideline to treat the RHCC after thermal ablation.

## Conclusions

In summary, the results of the present study suggested that adjuvant sorafenib after RFA was associated with a lower incidence of tumor recurrence and longer survival than RFA only for RHCC within Milan criteria. Further prospective and randomized controlled studies are needed to validate these findings.

## Supplementary Information

Below is the link to the electronic supplementary material.

Supplementary file1 (DOCX 214 kb)

## Data Availability

Data available from the authors upon reasonable request and with permission of four hospitals authority in China.

## Abbreviations

ALBI: Albumin–bilirubin
AFP: Alpha-fetoprotein
BCLC: Barcelona Clinic Liver Cancer
CT: Computed tomography
CI: Confidence interval
HCC: Hepatocellular carcinoma
HR: Hazard ratio
HBV: Hepatitis B virus
LR: Liver resection
MRI: Magnetic resonance imaging
MVI: Microvascular invasion
OS: Overall survival
PSM: Propensity score matching
RFA: Radiofrequency ablation
RHCC: Recurrent hepatocellular carcinoma
TACE: Transcatheter arterial chemoembolization
TFS: Tumor-free survival
