# Correction to: Sorafenib as adjuvant therapy following radiofrequency ablation for recurrent hepatocellular carcinoma within Milan criteria: a multicenter analysis

**DOI:** 10.1007/s00535-022-01910-7

**Published:** 2022-08-03

**Authors:** Qunfang Zhou, Xiaohui Wang, Ruixia Li, Chenmeng Wang, Juncheng Wang, Xiaoyan Xie, Yali Li, Shaoqiang Li, Xianhai Mao, Ping Liang

**Affiliations:** 1 Department of Interventional Ultrasound, Chinese PLA General Hospital, 28 Fuxing Road, Beijing, 100853 China; 2 Department of Hepatobiliary Surgery, Hunan Provincial People’s Hospital, The First Affiliated Hospital of Hunan Normal University, Changsha, 410002 Hunan Province China; 3 Department of Liver Surgery, Sun Yat-Sen University Cancer Center, Guangzhou, 510060 Guangdong China; 4 Department of Medical Ultrasonics, Institute of Diagnostic and Interventional Ultrasound, The First Affiliated Hospital of Sun Yat-Sen University, Guangzhou, 510060 China; 5 Department of Liver Surgery, The First Affiliated Hospital of Sun Yat-Sen University, Guangzhou, 510060 Guangdong Province China

## Correction to: J Gastroenterol 10.1007/s00535-022-01895-3

In the original publication of the article, the corresponding authors should be Xianhai Mao and Ping Liang as given in this correction and not Qunfang Zhou.

