# Supplementary material for: Sorafenib as adjuvant therapy following radiofrequency ablation for recurrent hepatocellular carcinoma within Milan criteria: a multicenter analysis
Source: J Gastroenterol. 2022 Jul 11;57(9):684–94. doi: 10.1007/s00535-022-01895-3 (PMC9392709; doi:10.1007/s00535-022-01895-3)
Supplement: Supplementary file 1 — Supplementary file1 (DOCX 214 kb) [file 535_2022_1895_MOESM1_ESM.docx]

**Sorafenib as adjuvant therapy following radiofrequency ablation for recurrent hepatocellular carcinoma within Milan criteria: a multicenter analysis**

**Sorafenib as adjuvant therapy following radiofrequency ablation for recurrent hepatocellular carcinoma within Milan criteria: a multicenter analysis**

Qunfang Zhou^1^, Xiaohui Wang^2^, Ruixia Li^1^, Chenmeng Wang^1^, Juncheng Wang^3^, Xiaoyan Xie^4^, Yali Li^1^, Shaoqiang Li^5^, Xianhai Mao^2#^, Ping Liang^1#^

**Authors’ affiliations:**

1. Department of Interventional Ultrasound, Chinese PLA General Hospital, 28 Fuxing Road, Beijing 100853, China.

2. Department of Hepatobiliary Surgery, Hunan Provincial People's Hospital (The First Affiliated Hospital of Hunan Normal University) Changsha, Hunan province, 410002, China.

3. Department of Liver Surgery, Sun Yat-sen University Cancer Center, Guangzhou, Guangdong, 510060, China.

4. Department of Medical Ultrasonics, Institute of Diagnostic and Interventional Ultrasound,

The First Affiliated Hospital of Sun Yat-sen University, Guangzhou, 510060, China.

5. Department of Liver Surgery, The First Affiliated Hospital of Sun Yat-sen University, Guangzhou, Guangdong Province, 510060, China.

**# Correspondence**：

Xianhai Mao：Department of Hepatobiliary Surgery, Hunan Provincial People's Hospital (The First Affiliated Hospital of Hunan Normal University) Changsha, Hunan province, 410002, China. [mxhaiszy@yahoo.com](mailto:mxhaiszy@yahoo.com).

Ping Liang: Department of Interventional Ultrasound, Chinese PLA General Hospital, 28 Fuxing Road, Beijing 100853, China. [liangping301@hotmail.com](mailto:liangping301@hotmail.com).

**Supplementary Figure 1**. Flow chart to select recurrent hepatocellular carcinoma (RHCC) patients within Milan criteria.

**Supplementary Figure 2**. Kaplan-Meier survival curves for patients with different risk score. (A) Tumor-free survival (TFS) curve of entire patients with low and high risk group. TFS of Sorafenib-RFA and RFA in low risk group (B) and high risk group (C). There was significant difference between RFA or RFA-sorafenib treatment in the low risk group (*P* =0.065) and the high risk group (*P* <0.001).

**Supplementary Table 1**. Repeat recurrence characteristics of patients after RFA for recurrent hepatocellular carcinoma (RHCC) between the two groups.

**Supplementary Table 2**. Analysis of clinicopathological characteristics impacting tumor-free survival (TFS) in entire patients with recurrent hepatocellular carcinoma (RHCC) after propensity score matching (PSM).

**Supplementary Table 3**. Sorafenib-related adverse events of patients in RFA-sorafenib group.


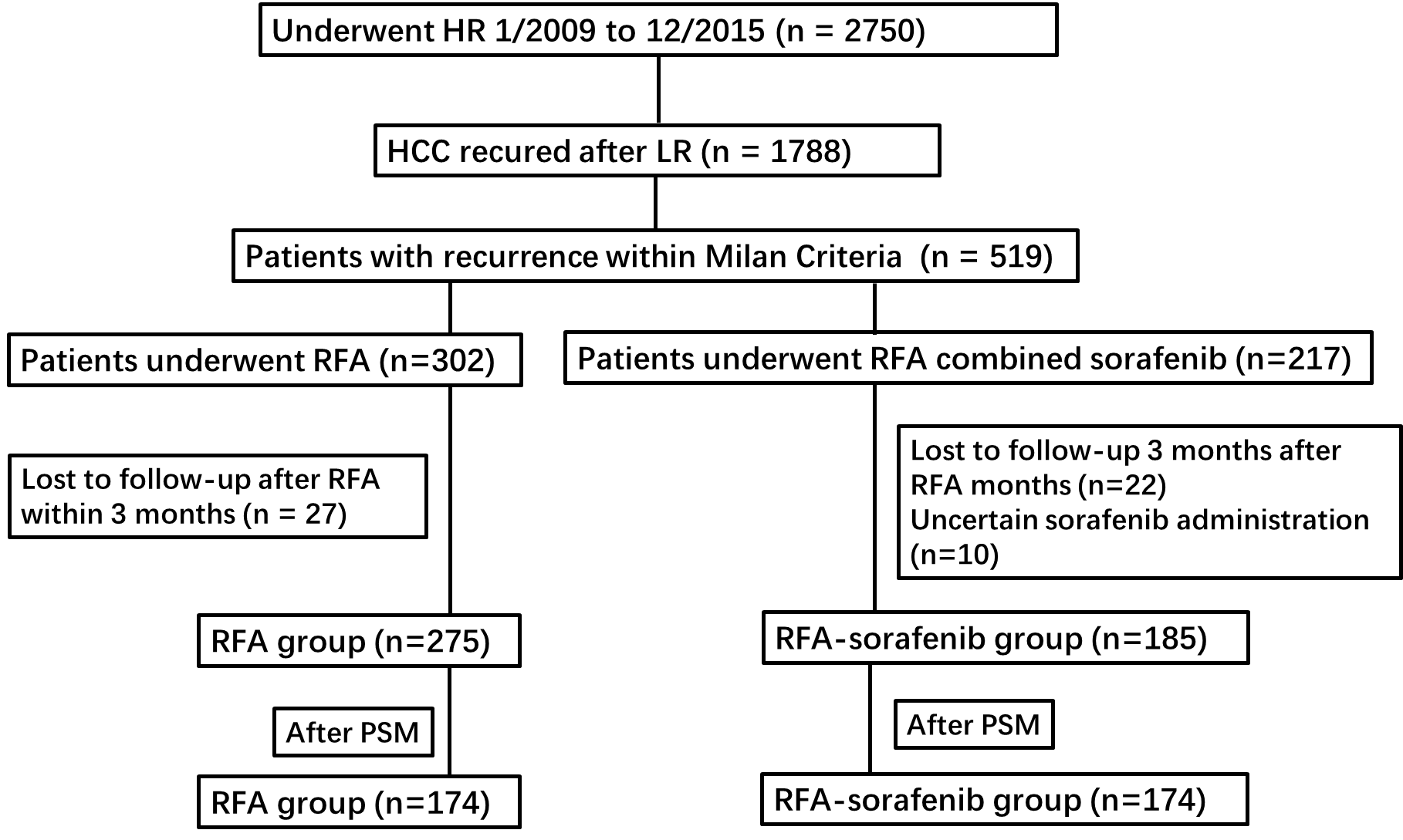


**Supplementary Figure 1**. Flow chart to select recurrent HCC patients within Milan criteria.

**Abbreviations**. LR: liver resection; HCC: hepatocellular carcinoma; RFA: radiofrequency ablation; PSM: propensity score matching.


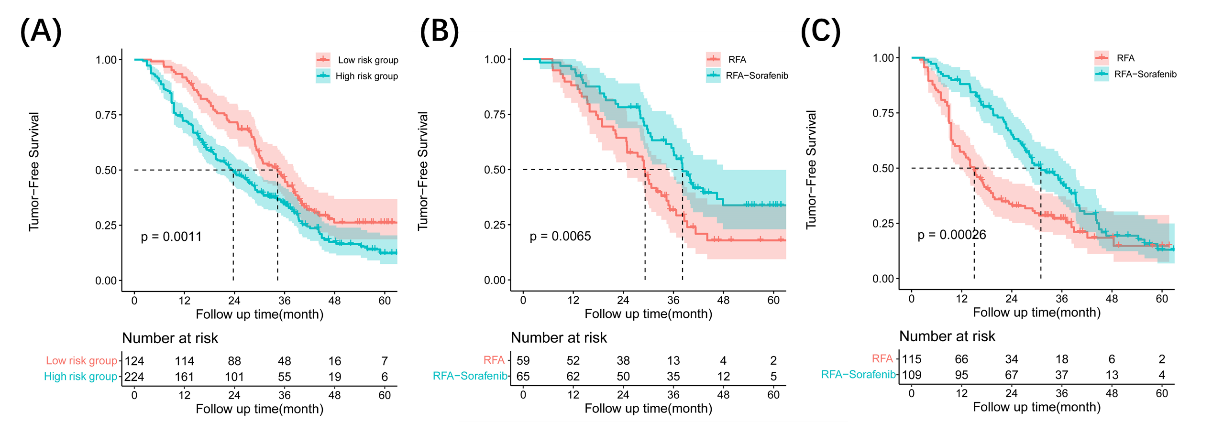


**Supplementary Figure 2**. Kaplan-Meier survival curves for patients with different risk score. (A) Tumor-free survival (TFS) curve of entire patients with low and high risk group. TFS of Sorafenib-RFA and RFA in low risk group (B) and high risk group (C). There was significant difference between RFA or RFA-sorafenib treatment in the low risk group (*P* =0.065) and the high risk group (*P* <0.001).

**Supplementary Table 1**. Repeat recurrence characteristics of patients after RFA for recurrent hepatocellular carcinoma (RHCC) between the two groups.

| **Variables** | **Before PSM** | | | **After PSM** | | |
| --- | --- | --- | --- | --- | --- | --- |
|  | RFA-sorafenib  (n=124) | RFA  (n=209) | *P* Value | RFA-sorafenib  (n=117) | RFA  (n=130) | *P*  Value |
| BCLC stage, n (%)  A  B  C | 59 (47.6)  38 (30.6)  27 (21.8) | 101 (48.3)  68 (32.5)  40 (19.2) | 0.288 | 57 (48.7)  34 (29.1)  26 (22.2) | 66 (50.8)  36 (27.7)  28 (21.5) | 0.948 |
| Number of HCC nodules  Single  Multiple | 53 (42.7)  71 (57.3) | 94 (45.5)  115 (54.5) | 0.691 | 51 (43.6)  66 (56.4) | 61 (46.9)  69 (53.1) | 0.599 |
| Maximum size  ≤3cm  > 3cm | 76 (61.3)  48 (38.7) | 131 (62.7)  78 (37.3) | 0.801 | 71 (60.6)  46 (39.3) | 81 (62.3)  49 (37.7) | 0.793 |
| Treatment pattern, n (%)  Re-resection  Ablation | 6 (5.0)  62 (50.0) | 4 (1.9)  99 (47.4) | 0.816 | 5 (4.3)  60 (51.3) | 2 (1.5)  59 (45.4) | 0.807 |
| TACE/HAIC | 16 (12.9) | 32 (15.3) |  | 14 (12.0) | 19 (14.6) |  |
| RT | 5 (4.0) | 8 ( 3.8 ) |  | 4 (3.4) | 3 (2.3) |  |
| TKI | 7 (5.6) | 12 (5.7) |  | 6 (5.1) | 7 (5.4) |  |
| ICI | 0 (0.0) | 2 (1.0) |  | 0 (0.0) | 1 (0.8) |  |
| TACE/HAIC+ TKI | 21 (16.9) | 41 (19.6) |  | 21 (17.9) | 30 (23.1) |  |
| TKI+ ICI | 5 (4.0 ) | 9 (4.3) |  | 5 (4.3 ) | 7 (5.4) |  |
| Other | 2 (1.6 ) | 2 ( 1.2) |  | 2 (1.7 ) | 2 (1.5) |  |

Note: other treatments include systemic chemotherapy and best supportive care. BCLC, Barcelona Clinic Liver Cancer; HAIC, hepatic arterial infusion chemotherapy; ICI, immune checkpoint inhibitor; RT, radiation therapy; TACE, transarterial chemoembolization; TKI, tyrosine kinase inhibitors.

**Supplementary Table 2**. Analysis of clinicopathological characteristics impacting tumor-free survival (TFS) in entire patients with recurrent hepatocellular carcinoma (RHCC) after propensity score matching (PSM).

|  | **Comparison** | **univariate analysis** | | **Multivariate analysis** | |
| --- | --- | --- | --- | --- | --- |
|  |  | **HR (95% CI)** | ***P*** | **HR (95% CI)** | ***P*** |
| **Recurrent stage data** | | | | | |
| Age level, years | <60 vs. ≥60 | 0.8 (0.63-1.0) | 0.064 |  |  |
| ALBI grade | I vs. II | **1.38 (1.06-1.96)** | **0.009** | **1.29 (1.14-1.91)** | **0.007** |
| HBV-DNA positive | No vs. yes | 0.87 (0.7-1.07) | 0.189 |  |  |
| Anti-virus | No vs. yes | 0.96 (0.78-1.18) | 0.696 |  |  |
| AFP level, ng/ml | <200 vs. ≥200 | **1.35 (1.05-1.73)** | **0.019** | 1.17 (0.94-1.45) | 0.163 |
| Tumor size. cm | ≤3 vs. >3 | 1.17 (0.88-1.55) | 0.277 |  |  |
| Tumor number | Single vs. multiple | **1.70 (1.30-2.21)** | **<0.001** | **1.65 (1.29-2.1)** | **<0.001** |
| Recurrent stage | Late vs. early | **2.12 (1.59-2.83)** | **<0.001** | **2.22 (1.75-2.85)** | **<0.001** |
| Types of treatment | RFA-Sorafenib vs. RFA | **1.76 (1.37-2.27)** | **<0.001** | **1.52 (1.22-1.89)** | **<0.001** |
| **Initial hepatectomy stage data** | | | | | |
| Primary tumor size, cm | ≤5  >5, <10  ≥10 | Reference  1.10 (0.86-1.41)  **2.0 (1.49-2.17)** | 0.443  **<0.001** | Reference  1.14 (0.89-1.46)  **1.83 (1.35-2.49)** | 0.315  **<0.001** |
| BCLC stage | A vs. B | **1.30 (1.04-1.63)** | **0.021** | **1.38 (1.09-1.73)** | **0.007** |
| MVI | Negative vs. positive | **1.56 (1.26-1.93)** | **<0.001** | **1.42 (1.14-1.77)** | **0.002** |
| Resection margin, cm | >1 vs. ≤1 | 0.9 (0.7-1.16) | 0.424 |  |  |
| Tumor differentiation | I-II vs. III-IV | 0.97 (0.78-1.2) | 0.780 |  |  |
| Tumor capsule | Complete vs. incomplete | 1.03 (0.92-1.14) | 0.651 |  |  |
| Hepatitis | No vs. yes | 0.92 (0.74-1.15) | 0.449 |  |  |
| Cirrhosis | No vs. yes | 0.96 (0.78-1.19) | 0.725 |  |  |

**Abbreviations**: ALBI, albumin-bilirubin; AFP, alpha-fetoprotein; BCLC, Barcelona Clinic Liver Cancer; HR, hazard ratio; CI: confidence interval; MVI, microvascular invasion.

**Supplementary Table 3**. Sorafenib-related adverse events of 185 patients in RFA-sorafenib group.

| **Adverse Events** | **All Events**  **N (%)** | **Grade 1–2 Events** | **Grade 3-4 Events** |
| --- | --- | --- | --- |
| Hand-foot skin reaction | 75 (40.5) | 71 (38.4) | 4 (2.1) |
| Hypertension | 62 (33.5) | 54 ( 29.2) | 8 ( 4.3) |
| Rash or desquamation | 41 (22.2) | 36 (19.5) | 5 (2.7) |
| Diarrhea | 36 (19.5) | 34 (18.4) | 2 (1.1) |
| Alopecia | 33 (17.8) | 41 (16.7) | 2 (1.1) |
| Fatigue | 32 (17.3) | 29 (16.2) | 2 (1.1) |
| Decreased appetite | 37 (20.0) | 35 (18.9) | 2 (1.1) |
| Voice change | 15 (8.1) | 15 (8.1) | 0 (0.0) |
| Oral mucositis | 14 (7.6) | 14 (7.6) | 0 (0.0) |
| Increased ALT/AST | 10 (5.4) | 9 (4.9) | 1 (0.5) |

**Abbreviations**: ALT, alanine aminotransferase; AST, aspartate aminotransferase.
